# Tick-Borne Encephalitis Virus Vaccine-Induced Human Antibodies Mediate Negligible Enhancement of Zika Virus Infection *In* *Vitro* and in a Mouse Model

**DOI:** 10.1128/mSphereDirect.00011-18

**Published:** 2018-02-07

**Authors:** James Duehr, Silviana Lee, Gursewak Singh, Gregory A. Foster, David Krysztof, Susan L. Stramer, Maria C. Bermúdez González, Eva Menichetti, Robert Geretschläger, Christian Gabriel, Viviana Simon, Jean K. Lim, Florian Krammer

**Affiliations:** a Department of Microbiology, Icahn School of Medicine at Mount Sinai, New York, New York, USA; b Graduate School of Biomedical Sciences, Icahn School of Medicine at Mount Sinai, New York, New York, USA; c Graduate School of Arts and Science, New York University, New York, New York, USA; d American Red Cross, Gaithersburg, Maryland, USA; e The Global Health Emerging Pathogens Institute, Icahn School of Medicine at Mount Sinai, New York, New York, USA; f Austrian Red Cross, Blood Service for Vienna, Lower Austria, and Burgenland, Vienna, Austria; g Ludwig Boltzmann Institute for Experimental and Clinical Traumatology, Graz, Austria; h Institute for Experimental Transfusion Medicine, Altenberg, Austria; i Divsion of Infectious Diseases, Department of Medicine, Icahn School of Medicine at Mount Sinai, New York, New York, USA; University of Maryland School of Medicine; Johns Hopkins Bloomberg School of Public Health; University of Texas Medical Branch

**Keywords:** ADE, TBEV, Zika, antibody-dependent enhancement, tick-borne encephalitis virus

## Abstract

Recent reports in the scientific literature have suggested that anti-dengue virus (DENV) and anti-West Nile virus (WNV) immunity exacerbates Zika virus (ZIKV) pathogenesis *in vitro* and *in vivo* in mouse models. Large populations of immune individuals exist for a related flavivirus (tick-borne encephalitis virus [TBEV]), due to large-scale vaccination campaigns and endemic circulation throughout most of northern Europe and the southern Russian Federation. As a result, the question of whether anti-TBEV immunity can affect Zika virus pathogenesis is a pertinent one. For this study, we obtained 50 serum samples from individuals vaccinated with the TBEV vaccine FSME-IMMUN (Central European/Neudörfl strain) and evaluated their enhancement capacity *in vitro* using K562 human myeloid cells expressing CD32 and *in vivo* using a mouse model of ZIKV pathogenesis. Among the 50 TBEV vaccinee samples evaluated, 29 had detectable reactivity against ZIKV envelope (E) protein by enzyme-linked immunosorbent assay (ELISA), and 36 showed enhancement of ZIKV infection *in vitro*. A pool of the most highly reacting and enhanced samples resulted in no significant change in the morbidity/mortality of ZIKV disease in immunocompromised *Stat2*^*−/−*^ mice. Our results suggest that humoral immunity against TBEV is unlikely to enhance Zika virus pathogenesis in humans. No clinical reports indicating that TBEV vaccinees experiencing enhanced ZIKV disease have been published so far, and though the epidemiological data are sparse, our findings suggest that there is little reason for concern. This study also displays a clear relationship between the phylogenetic distance between two flaviviruses and their capacity for pathogenic enhancement.

**IMPORTANCE** The relationship between serial infections of two different serotypes of dengue virus and more severe disease courses is well-documented in the literature, driven by so-called antibody-dependent enhancement (ADE). Recently, studies have shown the possibility of ADE in cells exposed to anti-DENV human plasma and then infected with ZIKV and also in mouse models of ZIKV pathogenesis after passive transfer of anti-DENV human plasma. In this study, we evaluated the extent to which this phenomenon occurs using sera from individuals immunized against tick-borne encephalitis virus (TBEV). This is highly relevant, since large proportions of the European population are vaccinated against TBEV or otherwise seropositive.

## Abstract (precis)

The relationship between serial infections of two different serotypes of dengue virus and more severe disease courses is well-documented in the literature, driven by so-called antibody-dependent enhancement (ADE). Recently, studies have shown the possibility of ADE in cells exposed to anti-DENV human plasma and then infected with ZIKV and also in mouse models of ZIKV pathogenesis after passive transfer of anti-DENV human plasma. In this study, we evaluated the extent to which this phenomenon occurs using sera from individuals immunized against tick-borne encephalitis virus (TBEV). This is highly relevant, since large proportions of the European population are vaccinated against TBEV or otherwise seropositive.

## INTRODUCTION

As the world becomes a more interconnected web of travel and commerce, there is a need to recognize the interaction of different endemic diseases in disparate regions. Much of this will take the form of studying how endemic diseases in some regions are affected by emerging pathogens in others. Of note, recent studies detailing how Zika virus (ZIKV) pathogenesis is enhanced by antiflavivirus humoral immunity both *in vitro* and in *in vivo* mouse models have created a need to understand how individuals in other regions with endemic flavivirus circulation and widespread vaccination might be affected (1, 2). The tick-borne encephalitis viruses (TBEVs), in particular, represent a growing public health threat throughout much of Europe and Asia, with an estimated 10,000 to 15,000 cases reported annually (3). The European subtype of TBEV also represents a modern medical success story, as vaccine coverage of an estimated 88% in Austria has led to a >90% reduction in the burden of disease (4–7). These historically high vaccination rates represented a pertinent cohort for study. However, high vaccination rates and a large population of seroconverted individuals are not exclusive to Austria: many European states (e.g., Germany [8], Switzerland [9], and Lithuania [10]) have high rates of immunity from vaccination. A strong economy combined with a trend toward globalization has also led to a high level of travel abroad in these countries (11). The possibility of imported Zika virus cases looms large. Past studies have shown that the ZIKV E protein and TBEV E are structurally similar (12), and various antibodies targeting specific epitopes on ZIKV E cross-react with the TBEV E protein (13). This represents a pertinent unanswered question: can immunity against TBEV enhance ZIKV disease? In this study, we evaluated the sera of 50 donors vaccinated with the inactivated FSME-IMMUN TBEV vaccine for cross-reactivity to ZIKV E protein by enzyme-linked immunosorbent assay (ELISA) and antibody-dependent enhancement (ADE) potential in Fcγ receptor (FcγR)-bearing human K562 myeloid cells. Samples positive for ELISA reactivity and ADE *in vitro* were pooled and passively transferred in our recently published *in vivo* model of ZIKV infection in immunocompromised *Stat2*^−/−^ mice. While immunocompromised mice have their limitations as a representative model of human disease (impaired interferon [IFN]-driven antiviral signaling, for example), we believe that they have relevance as a screening tool to evaluate whether or not further study is warranted. As we discovered, sera from donors vaccinated against TBEV induce weak enhancement of ZIKV infection *in vitro* but do not induce a significant level of enhancement in an *in vivo* model.

## RESULTS

**FIG 1:**
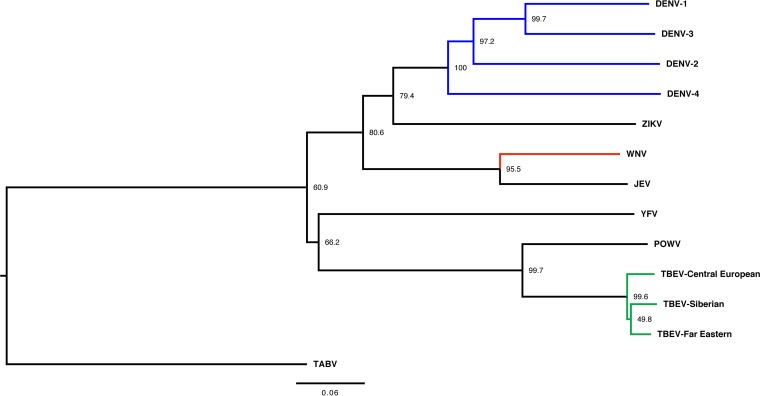
Phylogenetic tree of medically important flaviviruses based on E protein amino acid diversity. Node labels refer to bootstrap values as a percentage of 1,000 iterations, with the indicated taxa clustered together. The TBEV complex is shown in green, the DENV complex is shown in blue, and WNV is shown in red. TBEV and Powassan virus (POWV) are the only tick-borne viruses shown; all others are mosquito borne. YFV, yellow fever virus; WNV, West Nile virus; JEV, Japanese encephalitis virus. The tree was generated using the Clustal Omega algorithm, analyzed in Mega7 to generate bootstrap values (as a percentage) out of 1,000 iterations, visualized in FigTree, and rooted to Tamana bat virus (TABV) as the outgroup to show diversity among flaviviruses. The scale bar represents a 6% change in amino acids.

To investigate the possibility that TBEV cross-reactivity against ZIKV supports antibody-dependent enhancement, we first examined the phylogenetic relationship of these related flaviviruses (Fig. 1). When determined as a factor of E protein amino acid sequence similarity, the phylogenetic relationship between TBEV and ZIKV appears quite distant, in two different branches, with Japanese encephalitis virus (JEV), West Nile virus (WNV), and all four DENV serotypes being more closely related to ZIKV than the TBEV complex is. Also of note is the clustering of ZIKV with DENV, underscoring past descriptions of ZIKV as “the fifth serotype of dengue” (14). With this degree of similarity between ZIKV and TBEV, we speculated that TBEV vaccinee serum might be an effective test of the limits of ADE of ZIKV in *in vitro* and murine *in vivo* models.

**FIG 2:**
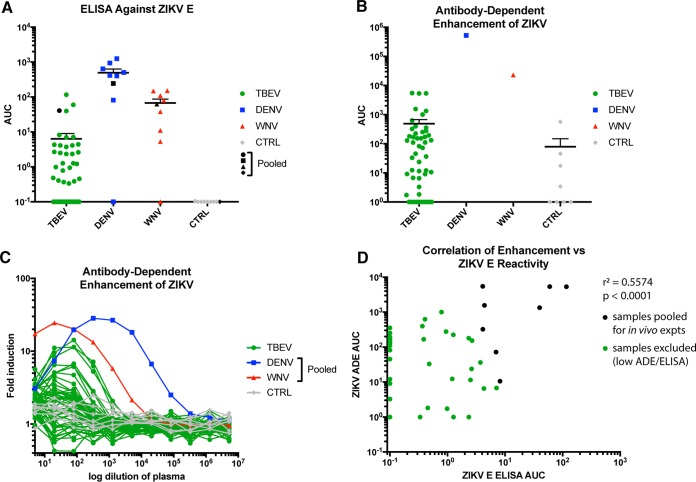
Anti-TBEV serum reacts to ZIKV E and enhances ZIKV infection *in vitro*. (A) AUC values from ELISAs against ZIKV E. Shown in blue are a preselected set of anti-DENV samples, with a range of reactivity. Shown in red are a similarly preselected set of anti-WNV samples. In gray are 8 naive-control (CTRL) plasma samples. In black for each are the pooled stocks used for later animal experiments. (B) AUC values derived from ADE *in vitro* experiments using ZIKV strain PRVABC59/2015 in K562 cells. DENV and WNV are plasma pools, as described for panel A. Naive-control samples are the same 8 samples employed to obtain the results shown in panel A. Lines represent geometric means. Error bars represent the standard errors of the means (SEM). (C) Raw ADE induction values derived from flow cytometry of K562 cells infected with ZIKV and incubated with the indicated serum/plasma sample. AUC values shown in panel B are based on this data. AUC values were calculated using all values up to the peak value for each curve. (D) Correlation analysis between ZIKV E ELISA AUCs and *in vitro* ADE AUCs. Each point represents one donor. *r*^2^ = 0.5574, *P* < 0.0001.

We first wanted to determine the contribution of *in vitro* mechanisms. To this end, anti-ZIKV E protein ELISAs were performed. As shown below, several of the TBEV vaccinee samples have area under the curve (AUC) values indicating reactivity. It is clear that anti-TBEV vaccination induces ZIKV cross-reactive humoral immunity (Fig. 2A). As expected, these vaccinee samples had much lower AUC values than pooled plasma from DENV- or WNV-infected individuals. This is most likely attributable to the phylogenetic relationships between TBEV, WNV, DENV, and ZIKV E protein (Fig. 1). A number of TBEV vaccinee samples also had low ELISA reactivity. This might have been the result of different responses to vaccination and donor heterogeneity. A similar breadth was also observed in anti-DENV and anti-WNV donor plasma.

**FIG 3:**
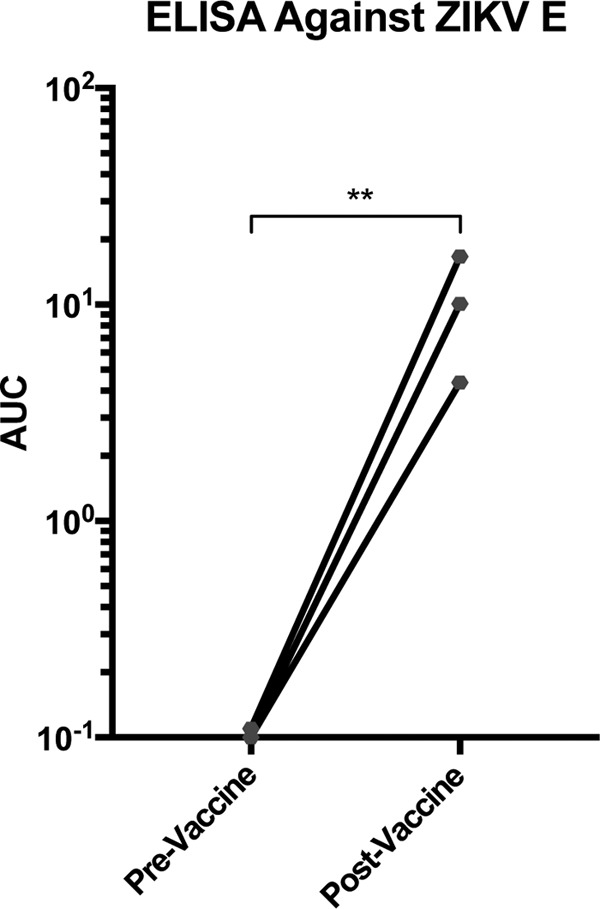
ZIKV E protein reactivity of three human TBEV vaccinees with serum harvested prevaccination and approximately 3 weeks postvaccination. ELISAs for ZIKV E protein were conducted as described in the legend of Fig. 1. **, *P* < 0.001 (ratio *t* test).

Reactivity to ZIKV E does not guarantee enhancement, since specific binding and functional mechanisms must be involved. Non-E-reactive antibodies might also drive ADE. To appropriately test for these mechanisms, TBEV vaccinee samples were evaluated in an *in vitro* assay of ZIKV infectivity in K562 cells expressing CD32 (FcγRII) (Fig. 2B and C). K562 cells are naturally nonpermissive to flaviviruses, but entry via virus-antibody complexes binding to FcγRII enables infection (15). Vaccinee samples had AUC values 10- to 20-fold higher on average than those of naive-control samples. The DENV and WNV pooled controls also induced a much larger amount of ADE than the TBEV vaccination samples and naive controls, as expected based on phylogenetic data. We were also interested in the extent to which the enhancement potential *in vitro* correlated with cross-reactivity against ZIKV E via ELISA. The statistical relationship between these two measures is considerable, implying a causative mechanism (Fig. 2D). However, there were a number of samples that lacked any measurable ZIKV E reactivity but had enhanced infectivity. In total, 28/50 samples (56%) had anti-ZIKV E reactivity via ELISA, while 40/50 (80%) enhanced ZIKV infectivity *in vitro*. Sixteen of forty samples enhanced infection without any detectable anti-ZIKV E reactivity. For these donors, humoral immunity against other ZIKV proteins may drive enhancement. Interestingly, matched pre- and post-TBEV vaccination samples from three volunteers demonstrated that anti-ZIKV cross-reactive immunity was induced to low levels by vaccination (Fig. 3).

**FIG 4:**
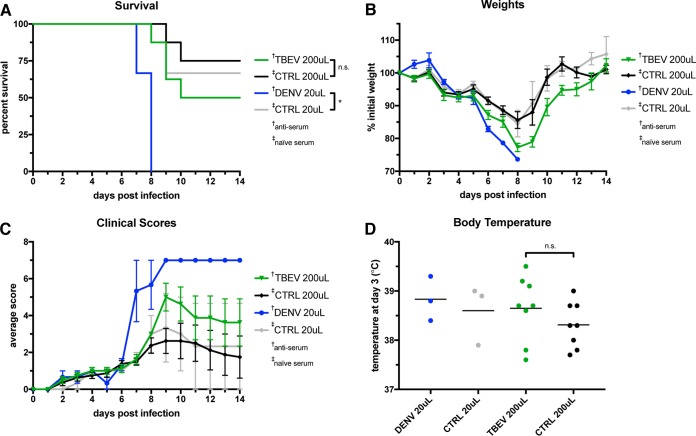
*In vivo* model of ZIKV enhancement in* Stat2*^*−/−*^ mice. Mice were given the indicated doses of naive-control, anti-TBEV, or anti-DENV serum/plasma (in 200 μl, total, with PBS as the diluent). In the 20-μl and 200-μl groups, 3 and 8 mice were tested, respectively. Results shown are from two separate experiments. (A) Kaplan-Meier survival curves. (B) Weight curves. On days 7 and 8, the anti-DENV group consisted of one mouse. (C) Average clinical symptom scores for each group, using a 6-point system and a score of 7 for deceased animals. (D) Rectal body temperatures measured on day 3. n.s., not significant; *, *P* < 0.05. Values represent geometric means. Error bars represent the SEM. †, antiserum was used; ‡, naive serum was used.

Given the nature of this *in vitro* data, we next tested TBEV vaccinee serum for signs of ADE in an *in vivo* model. In a previous study, *Stat2*^*−/−*^ mice showed severe signs of ZIKV disease when the virus was administered in concert with anti-DENV plasma with a large window of enhancement. Mice that received 20 μl of pooled anti-DENV plasma had a 20% rate of survival compared to >90% for animals that received pooled naive-control plasma (1). This window between experimental and control groups made *Stat2*^*−/−*^ mice an ideal platform to test our TBEV vaccinee samples. If anti-TBEV humoral immunity enhances ZIKV pathogenesis, this is the best model at our disposal to observe it in controlled settings. In dose escalation studies, 200 μl of anti-TBEV serum was chosen as the dose to elicit the largest amount of ADE (Fig. S1). As shown in Fig. 4A, animals that received 200 μl of pooled anti-TBEV serum survived at a rate (50%) that, while distinct from that of 200-μl naive controls (75%), does not rise to levels of likely physiological significance in humans. Mice receiving 200 μl of anti-TBEV serum also exhibited some pathogenesis in measures of morbidity, including weight loss and clinical symptom score (Fig. 4B and C). But again, no statistical significance between these and 200-μl naive-control animals was observed, indicating that there might be little physiological significance to these effects that could be translated to human disease states.

10.1128/mSphereDirect.00011-18.1 FIG S1 Dose escalation of ZIKV enhancement in* Stat2*^*−/−*^ mice. Mice were given the indicated doses of naive-control or anti-TBEV serum/plasma (in 200 μl, total, with PBS as the diluent). In the 20-μl and 200-μl groups, 3 and 8 mice, respectively, were tested. This figure includes data shown in Fig. 4; pooled results are shown from two separate experiments. (A) Kaplan-Meier survival curves; (B) weight curves; (C) average clinical symptom scores for each group; (D) rectal body temperatures detected on day 3. Download FIG S1, TIF file, 1.6 MB. Copyright © 2018 Duehr et al. 2018 Duehr et al. This content is distributed under the terms of the Creative Commons Attribution 4.0 International license.

We were also interested in the relationship between fever and other clinical symptoms. We previously observed the induction of fever in this model, with a markedly higher fever in anti-DENV animals (>38°C) than in controls (~37°C). Early-onset low-grade fever ranging from 37.8 to 38.5°C is a known clinical marker of Zika virus disease in humans, and high-grade fever (38.9°C to 41.7°C) is an early indicator of dengue hemorrhagic fever, a disease exacerbated by ADE (16). In the animals injected with anti-TBEV serum before ZIKV infection, we observed a low-grade fever (~38.6°C) on day 3 not significantly distinct from the fever observed in anti-DENV positive-control animals, ~38.8°C (Fig. 4D) (unpaired *t* test *P* = 0.7). A lower-grade fever was also observed in naive-control animals (~38.3°C).

## DISCUSSION

The ADE effect that increases viremia and disease pathogenesis between different dengue virus serotypes is well documented in the medical literature (17–19). Further, the finding that immunity against closely related flaviviruses can enhance flavivirus infection *in vitro* is not new (20). Epidemiological and mechanistic studies extending these phenomena to more complex systems, though, are sparse. Concerns about ZIKV-induced microcephaly of infants born to seropositive mothers in Brazil have reignited the drive to identify and characterize these phenomena as determinants of pathogenesis. In particular, a number of recent studies have highlighted the possibility that dengue virus immunity affects Zika virus pathogenesis (1, 2). Other studies have not observed this using models of serial infection in macaques and clinical data from DENV-seropositive ZIKV-infected cohorts of patients (21, 22). Mechanistic questions and low cohort sizes have stressed the need for more studies of an epidemiological origin. Even so, an important question is raised: is it possible that enhancement of ZIKV pathogenesis is induced by other flaviviruses? In this study, we provide evidence in *in vitro* and *in vivo* models that enhanced ZIKV infection is likely not induced by humoral anti-TBEV immunity induced by TBEV vaccination. We utilized serum samples taken from individuals recently vaccinated with FSME-IMMUN (Baxter/Pfizer), derived from the Central European subtype (Neudörfl strain). However, all three subtypes of TBEV (Central European, Siberian, and Far Eastern) are closely related based on sequence identity at the amino acid level (>94%) (23), and cross-protective immunity has been observed in animals vaccinated with the Central European subtype vaccine (24, 25). Also, the vast majority of serological clinical assays cannot distinguish between the subtypes due to cross-reactivity against E protein. For these reasons, we are confident that our findings with FSME-IMMUN will apply to other TBEV vaccines as well.

Our anti-TBEV samples came from vaccinees enrolled in programs beginning at an early age (the WHO recommends the vaccination for all persons ≥1 year old in countries where the disease is endemic) (5) and often including multiple boosters every 5 to 10 years (26). Our study utilizes samples drawn from participants between the ages of 23 and 34 years. This diversity of anti-ZIKV E titer is therefore unsurprising. It is likely that different individuals target different E protein epitopes, with some participants becoming increasingly less ZIKV reactive and others stabilizing or enriching their response against ZIKV-TBEV cross-reactive epitopes. For this and other reasons, we pooled only the most ZIKV E-reactive and ZIKV-enhancing serum samples for use *in vivo*.

Other groups have recently described that reactivity and enhancement do not share an entirely 1:1 correlation (27), and our findings here concur. Our prior studies showed that serum derived from macaques inoculated with the Yellow Fever virus (YFV) 17 D live attenuated vaccine failed to enhance ZIKV infectivity *in vitro* (1). Given the phylogenetic clustering of YFV and TBEV, this makes sense in the context of our findings with TBEV. Other mechanisms must play a role in whether or not serum, plasma, or monoclonal antibodies are enhancing. Ultimately, a mounting weight of evidence shows that phylogenetic distance between the inducing and enhanced viruses (and, by proxy, the reactivity of the induced polyclonal response against the enhanced virus) is predictive.

In dose-response studies of our samples *in vivo*, anti-TBEV serum showed the greatest amount of enhancement at the highest dose (200 μl). This is in contrast to what occurred with anti-DENV plasma, which had a protective effect at 200 μl. We previously theorized that this might be due to the relative phylogenetic distances of WNV and DENV from ZIKV. The four most common DENV serotypes are the most similar to ZIKV with regard to E protein amino acid sequence. WNV and TBEV, on the other hand, are much less closely related to ZIKV (or each other). Since more closely related viruses will often have more similar antigenic sites (28), it follows that higher doses of serum/plasma with more distantly related antigenic targets are required to neutralize and/or enhance virus.

In summary, our evidence shows that anti-TBEV immunity induces negligible enhancement of ZIKV disease in a murine *in vivo* model and is unlikely to cause enhanced ZIKV disease in humans. The question of the breadth of ADE has been a cause for concern to the emerging infectious disease field for some time, since increasingly more studies have shown induced ADE arising from further phylogenetic breadths. Our study shows a tapering of enhancement attributable to this phylogenetic diversity, which is cause for further examination.

## MATERIALS AND METHODS

### Phylogenetic tree.

E protein amino acid sequences (Table 1) were obtained from GenBank in FASTA format with a preference for whole-genome sequences partitioned into designated mature peptides. The sequences were aligned using the Clustal Omega Multiple Sequence Alignment (MSA) server maintained by the European Molecular Biology Laboratory’s European Bioinformatics Institute (EMBL-EBI). A phylogenetic tree was then generated using the maximum likelihood method based on the JTT matrix-based model in Mega7 (29). Node labels indicate bootstrap values as a percentage of trees with similarly clustered taxa out of 1,000 iterations. Visualization was done using FigTree v1.3.1 (Rambaut group, University of Edinburgh) and rooted to Tamana bat virus (TABV) as an outgroup to show relative diversity among other flaviviruses (Fig. 1).

**TABLE 1:** E protein amino acid sequences used to create the phylogenetic tree in Fig. 1

Tree label	Virus name	Strain/isolate	GenBank accession no.
DENV-1	Dengue virus 1	BR/1141_2011/AL/2010	AFD04702.1
DENV-2	Dengue virus 2	BR/SJRP/869/2013	AKQ00027.1
DENV-3	Dengue virus 3	United States/633798/1963	AFZ40124.1
DENV-4	Dengue virus 4	H241	ALB78116.1
TABV	Tamana bat virus	Tr127154	NP_776027.1
JEV	Japanese encephalitis virus	DH10M978	ALD09612.1
POWV	Powassan virus	P0375	AMY50382.1
TBEV-Far Eastern	Tick-borne encephalitis virus	Far Eastern/Tomsk-M202	AIL33470.1
TBEV-Siberian	Tick-borne encephalitis virus	Siberian/Kolarovo	ACN42746.2
TBEV-Central European	Tick-borne encephalitis virus	Central European/Neudörfl	NP_775503.1
WNV	West Nile virus	Lineage 2	NP_776014.1
YFV	Yellow fever virus	17 D vaccine	NP_740305.1
ZIKV	Zika virus	PRVABC59/2015	ANW07476.1

### Donor samples.

TBEV-vaccinated donor serum samples were provided by the Vienna Blood Center in Austria. All donors received an initial vaccination of FSME-IMMUN (Baxter/Pfizer) at a very young age (~1 to 3 years old), and most received subsequent boosters every 3 to 5 years. Samples tested in this study were obtained from male and female volunteers aged between 23 and 34 years (median, 29 years). None of the participants were vaccinated within 4 weeks of the blood draw. All samples were confirmed as WNV negative via PCR. Patients with past DENV infection were excluded based on health questionnaires completed at the time of blood collection. All samples were heat inactivated and filtered before use in this study.

All blood donors from Austria gave their informed consent for using residual test samples for scientific purposes. The responsible ethics commission of Upper Austria had no objection to the use of residual anonymized blood donor samples for research after informed consent was obtained.

Deidentified pre- and post-TBEV serum samples from the three vaccinated study participants were made available for research through the Personalized Virology Initiative (PVI), Icahn School of Medicine at Mount Sinai (institutional review board [IRB] number 1600772). Postvaccination samples were collected approximately 3 weeks after intramuscular vaccination with FSME-IMMUN (Baxter/Pfizer) as reported by the study participants.

DENV-infected blood donor plasma samples were selected from 270,049 samples donated to the American Red Cross in Puerto Rico between March 2010 and August 2013, as previously described (1). Initially, samples were screened via either DENV NS1 antigen ELISA or individual donation transcription-mediated amplification (TMA) testing (Gen-Probe, San Diego, CA). Donors were considered DENV infected only if both the initial and subsequent TMA test results were positive. Of 173 DENV-infected blood donors, we received 156 plasma units, of which 15 were ZIKV E reactive and used for the current study.

WNV-infected donor samples were identified via screening of 52,355,427 blood donations in the United States between August 2003 and December 2011. Initial screening was through WNV nucleic acid testing (NAT) as previously described (30). Blood donors were designated WNV infected only if both the initial and repeat WNV NAT samples were reactive and WNV-specific IgM and/or IgG testing results were positive upon either initial or follow-up testing. All tests for WNV antibodies were conducted by Abbot Laboratories (Abbot Park, IL) in 2003 to 2004 or by Focus Diagnostics (San Juan Capistrano, CA) in 2003 to 2011. We received a total of 471 WNV-infected donor samples that met the aforementioned criteria. Of these, we included only samples that had positive reactivity for WNV-specific IgG documented (*n =* 146). For all WNV- and DENV-infected donors, information pertaining to age, gender, and whether a doctor’s visit resulted from their infection was available.

Samples from 15 healthy blood donors obtained from the American Red Cross between April 2008 and August 2010 served as a negative-control population. These negative-control samples were screened for anti-WNV reactivity. Human sample testing was conducted under the following IRB protocols: dengue virus (Gen-Probe Procleix) clinical protocol number 2012-016 and WNV protocol number 2003-011.

### ZIKV envelope protein ELISA.

Briefly, E protein (Protein Sciences Corp., Meriden, CT) was applied to Immulon 4 HBX plates overnight at 4°C at a concentration of 2 μg/ml (50 μl/well) in coating buffer (0.1 M Na_2_CO_3_-NaHCO_3_, pH 9.4). After removal of the coating buffer, plates were washed three times with phosphate-buffered saline (PBS) (pH 7.4) with 0.1% Tween 20 (TPBS). Blocking buffer (TPBS plus 3% dry milk powder) was then added, and the plates were incubated for 1 h at room temperature (RT). After being decanted, heat-inactivated serum was serially diluted in the plates at a 1:3 dilution, starting with a 1:100 dilution in blocking buffer. Murine anti-flavivirus E monoclonal antibody (MAb) clone D1-4G2-4-15 (31) was used as a positive control, and a murine IgG2a MAb was used as a negative control, both to ensure standardized results. All control MAbs were also diluted 1:3 in the plates, with a 30-μg/ml starting concentration in blocking buffer. After a 2-h incubation period with samples, the plates were washed three times with TPBS. Secondary horseradish peroxidase conjugated to anti-human IgG antibody (Sigma; A0293, 1:3,000) diluted in blocking buffer was then added. After a 1-h incubation, plates were washed four times with TPBS and developed with SigmaFast *o*-phenylenediamine dihydrochloride (OPD; 100 μl per well; Sigma). After 10 min, the reaction was stopped with 3 M HCl. The optical density at 490 nm was read on a plate reader. Background levels were analyzed, and area under the curve (AUC) values were calculated using GraphPad Prism.

### *In vitro* assay of antibody-dependent enhancement of ZIKV infection.

To determine the level of *in vitro* ADE of ZIKV infection, serial dilutions of serum or plasma were suspended in Roswell Park Memorial Institute 1640 (RPMI 1640) medium supplemented with 10% fetal bovine serum (FBS), 2 mM l-glutamine, 10 U/ml penicillin, and 10 μg/ml streptomycin and combined with a suspension of ZIKV (at a multiplicity of infection of 1, strain PRVABC59, GenBank accession number KU501215.1). Plates were then incubated for 1 h at 37°C prior to the addition of K562 cells (5 × 10^4^) in 96-well U-bottom plates. After 2 days, cells were washed and then fixed with 4% paraformaldehyde (PFA) and permeabilized. Intracellular staining buffer consisted of PBS with 0.2% bovine serum albumin (BSA), 0.05% saponin, and anti-flavivirus E murine monoclonal antibody 4G2 (1 µg/ml hybridoma line D1-4G2-4-15). Intracellular staining was done for 1 h at RT. After being washed, cells were incubated with secondary goat anti-mouse IgG antibody conjugated to phycoerythrin (1 µg/ml; Invitrogen). ADE of ZIKV infection was measured using flow cytometry; the proportion of infected cells was determined using a FACSCalibur and analyzed with FlowJo2 version 10.1.r7. Areas under the curve were calculated using GraphPad Prism.

### *In vivo* model of ZIKV infection using *Stat2*^*−/−*^ mice.

All murine studies were conducted in an animal biosafety level 2 plus facility according to the guidelines of the Animal Care and Use Committee of the Icahn School of Medicine at Mount Sinai. Five- to eight-week-old *Stat2*^*−/−*^ mice (kindly provided by Christian Schindler) were injected intraperitoneally in the right flank with 200 or 20 µl (diluted to an overall total volume of 200 µl in PBS) of heat-inactivated 0.22-µm-filtered and pooled samples from either healthy-control, DENV, WNV, or TBEV samples. The 10 samples combined equally into pools were chosen based on their high reactivity and on enhancement observed *in vitro*. Two hours later, mice were injected intradermally with 5 × 10^3^ PFU of ZIKV (strain PRVABC59, GenBank accession number KU501215.1). Groups were composed of 3 to 8 mice each, with a balanced amount of males and females between groups whenever possible. An even distribution of mice from different litters was used whenever possible. Mice were then monitored daily for 14 days for metrics of survival, weight, body temperature, and clinical symptom score. Symptom scoring was conducted according to predefined criteria, with a maximum possible score of 6. A point was awarded for each symptom: impacted walking, unresponsiveness, left hind leg paralysis, right hind leg paralysis, left front leg paralysis, and right front leg paralysis. Deceased animals were awarded a score of 7. Body temperature was measured daily at the same time of day using a rectal thermometer (Braintree Scientific) after animals were anesthetized. Anesthetization was conducted using inhalation exposure to ~2 to 3% isoflurane for 30 s. Animals that showed a >25% weight loss or full paralysis were humanely euthanized.

### Data analysis.

AUC values were chosen as a means of representing reactivity and enhancement because AUC values represent a measure not only of the peak or trough of a curve’s signal but also of the entire curve. Because of this, comparisons of these values are less prone to error. AUC values were calculated as follows: ELISA or ADE values at a range of serial dilutions were entered into GraphPad Prism and subjected to nonlinear regression. For ADE values, only dilutions up to and including the peak were taken into account. An integral was then calculated on the curve, giving the AUC (https://www.graphpad.com/guides/prism/7/statistics/index.htm?stat_area_under_the_curve.htm). As a value, the AUC incorporates both the overall height of the curve and the overall width (how many dilutions still retain detectable signal above background). In this way, the AUC incorporates the endpoint titer; the two measures should be very tightly correlated. In the AUC graphs presented in Fig. 2, dots represent individual samples, a line represents the geometric mean of the group tested, and error bars represent standard errors of the means (SEM).

The *in vivo* experiments in this study were not conducted in a blind manner. Except where indicated, statistical tests were performed using GraphPad Prism software. All intergroup comparisons *in vitro* and *in vivo* were conducted using the Student *t* test statistic, with a baseline threshold *P* of <0.05. Comparisons made between three or more groups were evaluated against an adjusted *P* value using the Bonferroni correction. All *t* tests were unpaired, except for those conducted to evaluate the data presented in Fig. 3. Since information was matched for each anonymized subject pre- and postvaccination in Fig. 3, we conducted a ratio *t* test. This method compares the geometric means of the ratios of the pre- and postvaccination values in a paired manner, more effectively showing the statistical significance of induced antibody titers.

### Data availability.

The data that support the findings of this study are available from the corresponding authors upon request.
