# Supplementary figures and images for: Tick-Borne Encephalitis Virus Vaccine-Induced Human Antibodies Mediate Negligible Enhancement of Zika Virus Infection In Vitro and in a Mouse Model
Source: mSphere. 2018 Feb 7;3(1):e00011-18. doi: 10.1128/mSphereDirect.00011-18 (PMC5806211; doi:10.1128/mSphereDirect.00011-18)

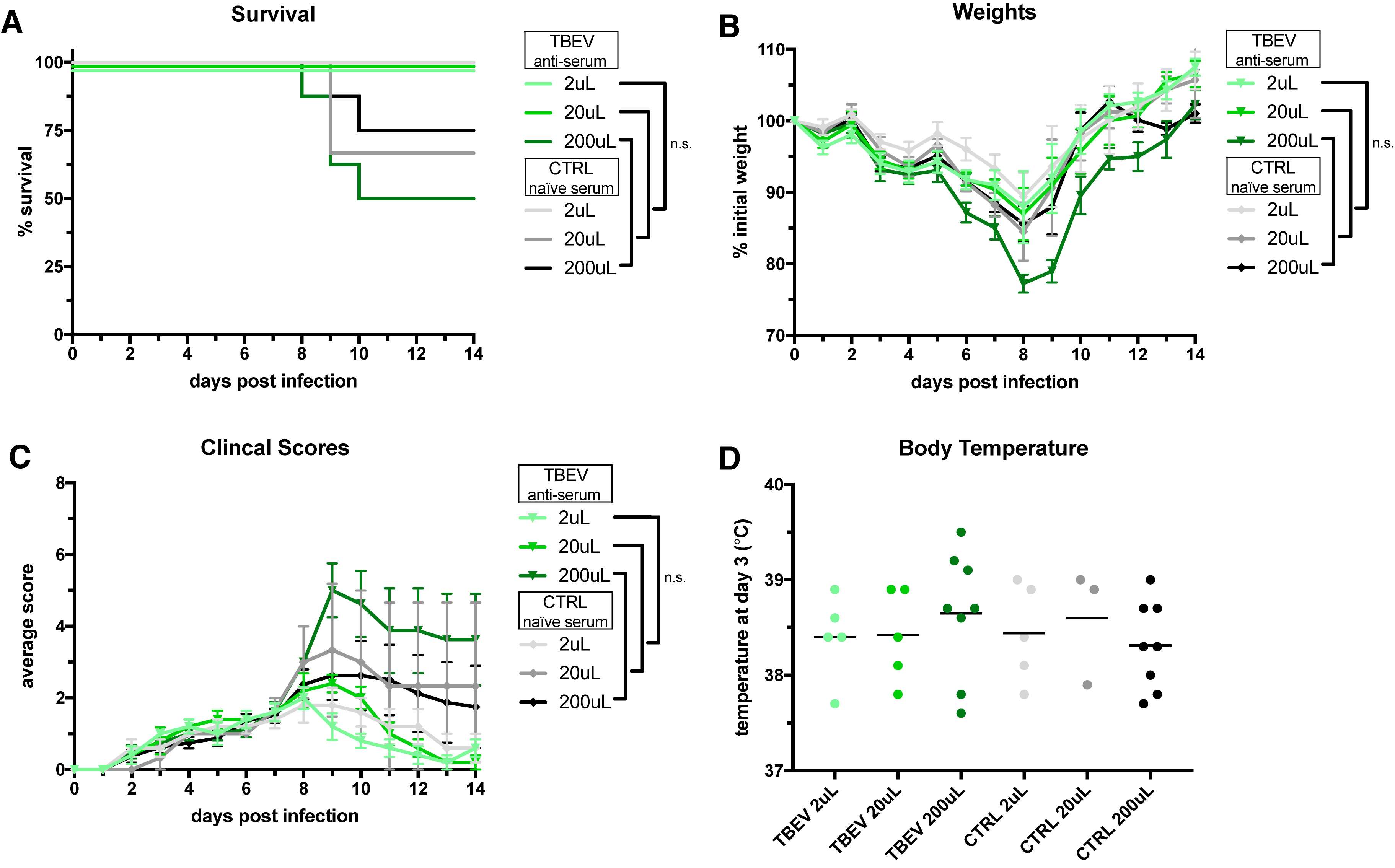

Supplement: FIG S1 [file sph001182473sf1.tif]
